# Bleomycin-Induced Pulmonary Fibrosis in Transgenic Mice Carrying the Human *MUC5B* rs35705950 Variant

**DOI:** 10.3390/cells13181523

**Published:** 2024-09-11

**Authors:** Suphachai Tharavecharak, Hajime Fujimoto, Taro Yasuma, Corina N. D’Alessandro-Gabazza, Masaaki Toda, Atsushi Tomaru, Haruko Saiki, Mei Uemura, Yurie Kogue, Toshiyuki Ito, Kazuki Furuhashi, Tomohito Okano, Atsuro Takeshita, Kota Nishihama, Ryoichi Ono, Osamu Hataji, Tetsuya Nosaka, Tetsu Kobayashi, Esteban C. Gabazza

**Affiliations:** 1 Department of Immunology, Faculty and Graduate School of Medicine, Mie University, Edobashi 2-174, Tsu 514-8507, Mie, Japan; 2 Department Pulmonary and Critical Care Medicine, Faculty and Graduate School of Medicine, Mie University, Edobashi 2-174, Tsu 514-8507, Mie, Japan; 3 Microbiome Research Center, Mie University, Edobashi 2-174, Tsu 514-8507, Mie, Japan; 4 Department of Diabetes, Endocrinology and Metabolism, Faculty and Graduate School of Medicine, Mie University, Edobashi 2-174, Tsu 514-8507, Mie, Japan; 5 Carl R. Woese Institute for Genomic Biology, University of Illinois at Urbana–Champaign, Urbana, IL 61801, USA; 6 Department of Microbiology and Molecular Genetics, Mie University Graduate School of Medicine, Tsu 514-8507, Mie, Japan; 7 Respiratory Center, Matsusaka Municipal Hospital, Tonomachi1550, Matsusaka 515-8544, Mie, Japan

**Keywords:** MUC5B, rs35705950, pulmonary fibrosis, inflammation

## Abstract

Idiopathic pulmonary fibrosis (IPF) is a progressive, often fatal lung disease characterized by tissue scarring and declining lung function. The *MUC5B* promoter polymorphism rs35705950, a significant genetic predisposition for IPF, paradoxically associates with better survival and slower disease progression than other IPF genotypes. This study investigates the potential paradoxical protective effects of this *MUC5B* variant in lung fibrosis. For this purpose, we developed a transgenic mouse model overexpressing the human *MUC5B* rs35705950 variant in the proximal large airways. Lung fibrosis was induced through subcutaneous injection of bleomycin. Results demonstrated significantly reduced lung fibrosis severity in transgenic mice compared to wild-type mice, assessed by trichrome staining, Ashcroft scoring, and hydroxyproline levels. Additionally, transgenic mice showed significantly lower levels of inflammatory cells and cytokines (TNFα, IL-6, IFNγ) and growth factors (PDGF, CTGF, IL-13) in the bronchoalveolar lavage fluid and lung tissues. There was also a significant decrease in mRNA expressions of fibrosis-related markers (periostin, fibronectin, Col1a1). In summary, this study reveals that mucin overexpression related to the *MUC5B* rs35705950 variant in the large airways significantly attenuates lung fibrosis and inflammatory responses in transgenic mice. These findings suggest that the rs35705950 variant modulates inflammatory and fibrotic responses in the proximal airways, which may contribute to the slower disease progression observed in IPF patients carrying this variant. Our study offers a possible explanation for the paradoxical beneficial effects of the *MUC5B* variant despite its role as a significant predisposing factor for IPF.

## 1. Introduction

Idiopathic pulmonary fibrosis (IPF) is a progressive and often fatal lung disease characterized by the scarring of lung tissue, leading to a gradual decline in lung function [1,2]. Despite extensive research, the pathogenesis of IPF remains poorly understood, and effective treatments are limited. Genetic and environmental factors that have been suggested to be involved in the development and progression of idiopathic pulmonary fibrosis include genetics, aging, sex, the lung microbiome, co-morbidities, the levels of some proteases such cathepsin B, cigarette smoking, environmental exposures, and air pollution [3,4,5]. A significant genetic factor in IPF development is the *MUC5B* gene, particularly the promoter polymorphism rs35705950, which contains a mutant T allele [6,7,8,9,10]. This variant has been identified as a strong predisposing factor for both sporadic and familial pulmonary fibrosis, with carriers showing an increased risk of developing the disease [6,7,8]. The mechanism behind this increased susceptibility is not fully understood, but evidence suggests that overexpression of MUC5B by distal bronchiolar and alveolar epithelial cells in IPF patients may lead to mucociliary dysfunction, promoting the development of pulmonary fibrosis in experimental animals [8,11,12]. Consistent with this observation, a recent study found that the distal airways in IPF patients differ structurally and dynamically from normal airway epithelia [13]. A delayed jamming transition in IPF airways leads to a prolonged unjammed phase, which is associated with the activation of collagen-producing mesenchymal cells. This extended unjammed phase correlates with aberrant activation of the Erb-B2 Receptor Tyrosine Kinase (ERBB) family of receptors and the Yes-Associated Protein (YAP) [13]. This signaling pathway interacts with the *MUC5B* promoter variant rs35705950 to induce mucin hypersecretion, implicating this variant in the pathogenesis of IPF [13].

Interestingly and somewhat paradoxically, IPF patients carrying the *MUC5B* promoter variant rs35705950 have a better prognosis, less progressive disease, and a lower lung bacterial burden than non-carriers [10,14,15,16,17]. In addition, studies have shown that familial interstitial pneumonia patients with the *MUC5B* variant exhibit less severe pathological alterations, and IPF patients with the variant experience a slower decline in forced vital capacity [18,19]. These counterintuitive observations have sparked considerable interest within the scientific community, suggesting that the *MUC5B* variant might confer some protective effects despite its association with increased disease susceptibility. Under physiological conditions, *MUC5B* encodes a gel-forming mucin protein essential for maintaining the mucociliary clearance system of the respiratory tract [20,21]. The overexpression of MUC5B, driven by the rs35705950 variant, may enhance mucociliary clearance and airway defenses against lung pathogens. Indeed, there is documented evidence that the MUC5B variant protects against severe COVID-19-associated acute lung injury [22,23].

Given these considerations, the mutant T allele of the *MUC5B* rs35705950 variant appears to contribute to a distinct IPF phenotype, wherein mucin overexpression is associated with both deleterious and protective outcomes. Recent studies have indicated that the dysfunction of mucociliary clearance resulting from aberrant mucin expression in the alveolar-bronchiolar or distal airways plays a role in the pathogenesis of IPF. Nevertheless, the specific mechanisms underlying the protective effects of the *MUC5B* rs35705950 variant in IPF remain elusive. In this investigation, we hypothesize that mucin overexpression in the proximal airways may confer protection against pulmonary fibrosis. To test this hypothesis, we developed a transgenic mouse model that overexpresses the human *MUC5B* rs35705950 variant, with the mutant T allele in the proximal bronchi. The objective of this study was to compare the development of fibrosis between these transgenic mice and wild-type controls. Notably, this study represents the first to engineer a transgenic mouse model overexpressing the human *MUC5B* rs35705950 variant in the proximal large airways and to demonstrate that mucin overexpression linked to the MUC5B promoter polymorphism rs35705950, a significant genetic risk factor for idiopathic pulmonary fibrosis (IPF), paradoxically exerts protective effects against lung fibrosis and inflammation.

## 2. Materials and Methods

### 2.1. Experimental Animals

Male and female WT C57BL/6J mice, sourced from Nihon SLC in Hamamatsu, Japan, weighed between 20 and 22 g and were 8 to 9 weeks old. They were given a two-week acclimation period before the experiments began. The mice were kept in a specific pathogen-free environment at 21 °C with a 12-h light/dark cycle in the Experimental Animal House at Mie University. Their cages were equipped with wood-wool nesting material, and they had unlimited access to water and food.

### 2.2. Generation of Human MUC5B [rs35705950] Transgenic Mice

**Figure 1:**
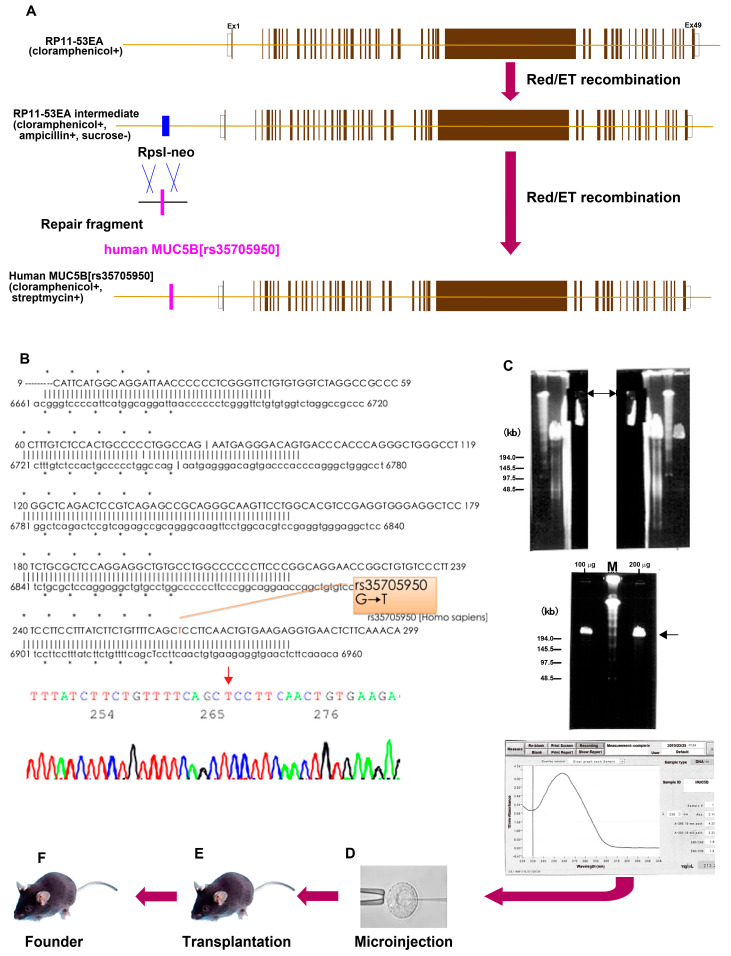
Generation of human MUC5B [rs35705950] transgenic mice. Preparation of a construct of human MUC5B [rs35705950] recombinant bacterial artificial chromosome (BAC) using the Red/ET recombination strategy (**A**). Sequence analysis of the junction DNA of the recombinant BAC clone (**B**). The expression construct linearized with PI-SceI (Proteinase Intein-Scel Endonuclease I) was separated by pulsed-field gel electrophoresis (**C**). The gel containing the expression construct within the agarose gel was cut out without UV irradiation (upper figure of panel **C**). The DNA fragments purified by electrophoretic elution and dialysis were applied to pulsed-field gel electrophoresis to confirm that the long-chain DNA fragments were purified without fragmentation (middle figure of panel **C**). The DNA concentration was determined using a NanoDrop spectrophotometer (Shimazu biotech, Kyoto, Japan) lower figure of panel **C**). The linearized recombinant BAC clone was microinjected into fertilized eggs of the C57BL/6J strain and the fertilized eggs were transplanted into the oviducts of pseudo-pregnant mice to get the founders (**D**–**F**). The marker used was M: NEB Low Range PFG marker. Red/ET, Red recombinase system (RedαRedβ proteins)/Electroporation-Transformation-cloning. Arrows indicate the bands.

To generate the human MUC5B [rs35705950] transgenic mice, we prepared a genomic clone encompassing the human *MUC5B* gene locus and constructed a recombinant bacterial artificial chromosome (BAC) clone by incorporating the *MUC5B* [rs35705950] sequence in-frame within its coding sequence. We identified and obtained the genomic clone containing the human *MUC5B* gene locus, designated RP11-532E4 (Figure 1A). Initially, a selection marker was inserted into the promoter region of the *MUC5B* gene within the human BAC clone using BAC recombination-mediated genetic engineering (Red/ET reaction). Subsequently, the mutation site within the promoter region of the *MUC5B* gene locus in the human BAC clone was deleted to prepare an intermediate form of the human *MUC5B* [rs35705950] recombinant BAC.

We then synthesized a human *MUC5B* [rs35705950] recombinant BAC repair fragment containing flanking sequences surrounding the selection marker insertion site and the single nucleotide substitution (G→T) rs35705950 in the promoter region. The selection marker inserted into the *MUC5B* promoter region was precisely replaced and removed using the human *MUC5B* [rs35705950] recombinant BAC repair fragment through further BAC recombination-mediated genetic engineering. Utilizing the Red/ET recombination strategy, we successfully constructed a human *MUC5B* [rs35705950] recombinant BAC without altering the exon/intron structure or the expression regulatory sequences of the *MUC5B* gene locus (Figure 1A) [24]. A sequence analysis of the junction DNA sequence of the recombinant BAC clone confirmed the correct insertion of the human *MUC5B* gene into the designated locus (Figure 1B).

The circular recombinant BAC clone was linearized using a specific restriction enzyme, and its long-chain DNA was purified through pulse-field gel electrophoresis (Figure 1C). The purity and concentration of the purified expression constructs were assessed using analytical agarose gel electrophoresis, which confirmed the successful recovery of pure, long-chain expression constructs with a DNA concentration of 213.2 ng/μL, sufficient for the creation of TG mice. The linearized recombinant BAC clone was then microinjected into fertilized eggs of the C57BL/6J strain to generate transgenic mouse founders. Embryos injected with the human *MUC5B* [rs35705950] recombinant BAC expression construct via microinjection into fertilized eggs from C57BL/6J mice were transplanted into the oviducts of pseudo-pregnant mice (Figure 1D,E) [25].

As a result, out of 305 fertilized eggs that survived after construct microinjection, 300 injected embryos were successfully transplanted into the pseudo-pregnant mice. A total of 377 fertilized eggs were injected with the expression construct, and 300 of these injected embryos were transplanted into pseudo-pregnant mice, resulting in the natural birth of 81 mouse offspring. All 74 individuals were successfully raised to weaning. Southern blotting screening by hybridization of genomic DNA fragments extracted from the offspring was performed using a [^32^P]-labeled probe. Three transgenic mouse founders were identified (Figure 1F). The number of copies of the expression construct introduced into these transgenic mouse founders ranged from one to three copies.

### 2.3. Pulmonary Fibrosis Induction

Pulmonary fibrosis was induced in the mice using BLM sourced from Nihon Kayaku, Tokyo, Japan. We implanted subcutaneous osmotic minipumps (Alzet model 2001; Durect Corporation, Cupertino, CA, USA), which administered BLM at a continuous rate of 1 µL/hour for seven days, totaling a dose of 100 mg/kg of mouse body weight, dissolved in 200 µL saline [26]. Control mice received identical pumps dispensing saline. The experimental setup included WT mice treated with either saline (WT/SAL, n = 8) or BLM (WT/BLM, n = 18), and hMUC5B TG mice treated with saline (h-rs35705950-TG/SAL, n = 10) or BLM (h-rs35705950-TG/BLM, n = 16).

### 2.4. Collection of BALF and Peripheral Blood

Mice were anesthetized with 3% isoflurane and BALF was collected as previously described [27]. The fluid was centrifuged at 1000 g for 10 min at 4 °C to pellet the cells, and the supernatant was stored at −80 °C for subsequent analyses. Total and differential cell counts were performed using a nucleocounter from ChemoMetec, Allerod, Denmark, and cells were stained with May-Grünwald Giemsa stain (Merk, Darmstadt, Germany).

### 2.5. Lung Tissue Collection

After euthanasia by an overdose of 5% isoflurane for over 60 s, lungs were harvested post-confirmation of death by cardiorespiratory arrest. One lung was fixed in formalin for histopathological analysis, including staining with hematoxylin-eosin and Masson’s trichrome, performed at Kinkiyoken Corporation, Otsu City, Shiga, Japan. The Ashcroft fibrosis score was used to assess fibrosis severity, with eight blinded observers scoring multiple microphotographs from each mouse [28]. The collagen area stained with Masson’s trichrome was quantified using WinRoof image processing software version 2015 (Mitani Corp., Fukui, Japan).

### 2.6. Biochemical Analysis

We measured the concentrations of TNFα, MCP-1, IL-6, IFN-γ, IL-13 (BD Bioscience, BD opt-EIA kits, San Diego, CA, USA), osteopontin (R&D Systems, Minneapolis, MN, USA), SP-D (Sino Biologicals, Beijing, China). The level of PDGF was measured using anti-PDGF (Genzyme, Boston, MA, USA) and biotin-labeled anti-PDGF antibodies. CTGF (abcepta, San Diego, CA, USA) was measured using commercial immunoassay kits according to the manufacturer’s protocols. Collagen type I was measured by an enzyme immunoassay using anti-collagen type I antibodies and anti-collagen type I biotin-conjugated antibodies from Rockland Immunochemicals Incorporated (Limerick, PA, USA). Lung hydroxyproline content was measured via a colorimetric assay (BioVision, San Francisco, CA, USA).

### 2.7. Immunohistochemical Staining

The MUC5B staining was performed using a polyclonal anti-MUC5B antibody (Proteintech, Rosemont, IL, USA) that cross-reacts with mouse MUC5b. This procedure was conducted at MorphoTechnology Corporation in Sapporo, Hokkaido, Japan, following standard protocols.

### 2.8. Gene Expression Analysis

Total RNA was extracted from lung tissues using Trizol Reagent (Invitrogen, Carlsbad, CA, USA) and reverse-transcribed with Superscript Reverse Transcriptase and oligo-dT primers (Invitrogen). Gene-specific primer pairs were used to amplify cDNA, with amplification conditions optimized for specificity and efficiency. The primers are described in Table 1. PCR products were visualized on a 2% agarose gel stained with ethidium bromide, and gene expression was normalized to glyceraldehyde 3-phosphate dehydrogenase (GAPDH).

### 2.9. Statistical Analysis

Data are presented as mean ± standard deviation (SD). The normality of the data distribution was assessed using the Kolmogorov-Smirnov test. The statistical differences between groups were evaluated using one-way ANOVA followed by the post hoc Newman-Keuls test for pairwise comparisons, selected for its sensitivity in detecting differences among multiple groups. All of the statistical analyses were performed using GraphPad Prism version 9.0 (GraphPad Software, San Diego, CA, USA), with statistical significance set at *p* < 0.05.

## 3. Results

### 3.1. Development of a Transgenic Mouse Model Expressing the Human MUC5B rs35705950 with the Mutant T Allele

**Figure 2:**
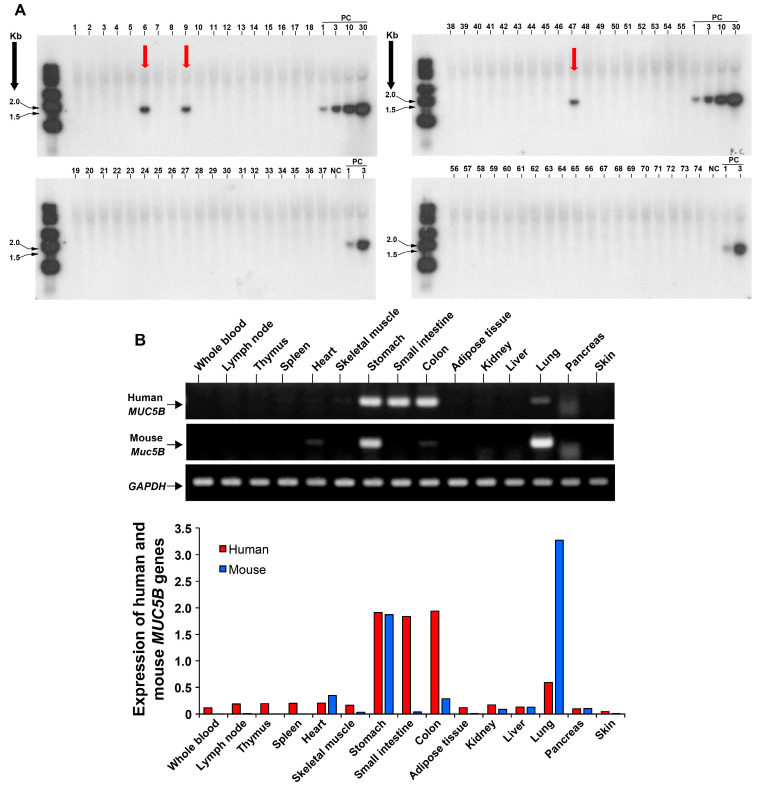
Expression of the human *MUC5B* [rs35705950] transgene. Southern blot screening for the transgene in the offspring, identifying three transgenic mouse founders (red arrows in (**A**)). Analysis of relative gene expression of the human *MUC5B* [rs35705950] transgene in various tissues and organs by PCR (**B**).

We generated a transgenic mouse model that overexpresses the full-length human *MUC5B* gene containing the T allele of the rs35705950 mutation. Initially, a genomic clone spanning the human *MUC5B* locus was prepared. A recombinant bacterial artificial chromosome (BAC) clone was then engineered by inserting the *MUC5B* [rs35705950] sequence in-frame within its coding sequence. Utilizing BAC recombination-mediated genetic engineering, a selection marker was integrated into the promoter region of the *MUC5B* gene within this human BAC clone (Figure 1A) [24]. Subsequently, a human *MUC5B* [rs35705950] recombinant BAC repair fragment was synthesized, incorporating flanking sequences around the selection marker insertion site and the single nucleotide substitution (G→T). The selection marker was then excised and replaced by the recombinant BAC repair fragment containing the T allele through further BAC recombination-mediated genetic engineering, thereby preserving the exon/intron structure and the regulatory sequences of the *MUC5B* gene locus (Figure 1A). Sequence analysis verified the precise integration of the human *MUC5B* gene at the intended locus within the recombinant BAC clone (Figure 1B). The BAC clone was linearized, and its long-chain DNA was purified using pulse-field gel electrophoresis (Figure 1C). The purified recombinant BAC DNA was microinjected into fertilized eggs of the C57BL/6J mouse strain, generating transgenic mouse founders (Figure 1D–F) [25]. Successful germline transmission of the BAC transgenic construct was confirmed via Southern blot analysis (Figure 2A). Both male and female transgenic mice exhibited high human *MUC5B* [rs35705950] gene expression levels by RT-PCR (Figure 2B).

### 3.2. Significant Expression of MUC5B in the Proximal Bronchial Segments in Human MUC5B rs35705950 Transgenic Mice with Lung Fibrosis

**Figure 3:**
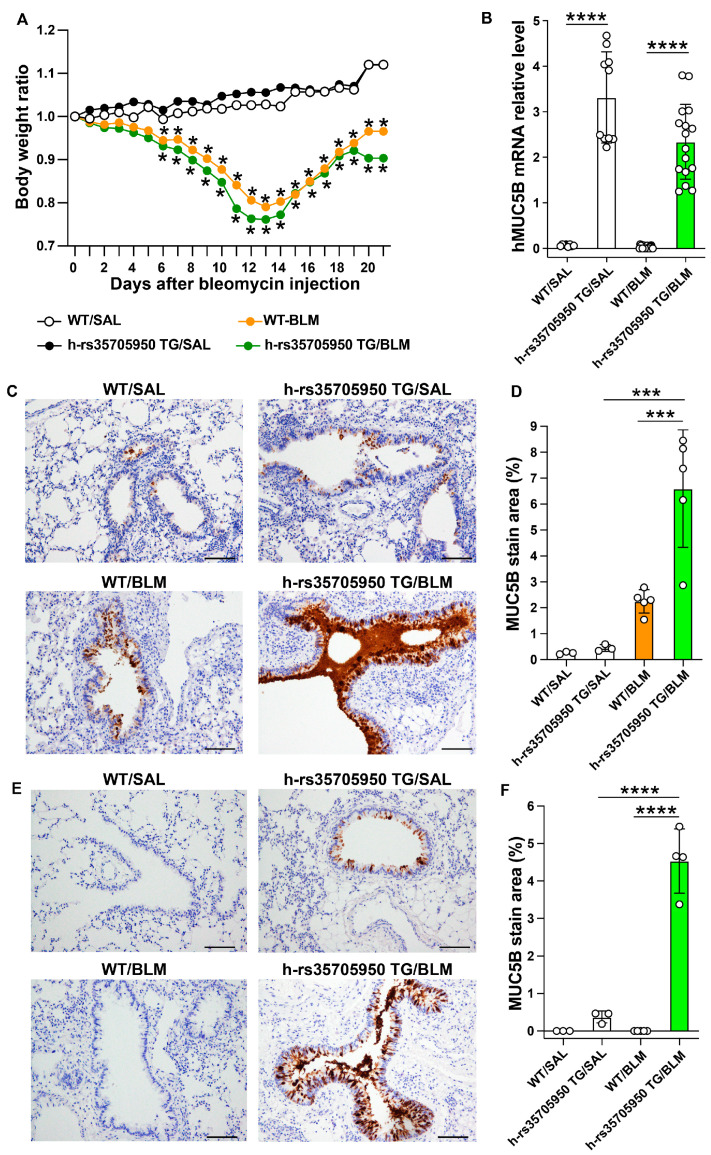
Sequential body weight changes and significant expression of the human *MUC5B* transgene rs35705950 in the proximal airways in the bleomycin-induced lung fibrosis model. Lung fibrosis was induced in wild-type (WT/BLM) and human MUC5B rs35705950 transgenic (h-rs35705950-Tg/BLM) mice through continuous subcutaneous administration of BLM. Control groups, consisting of WT (WT/SAL) and human MUC5B rs35705950 transgenic (h-rs35705950-Tg/SAL) mice, similarly received sterile physiological saline. Sequential body weight changes in the four groups of mice (**A**). Relative mRNA expression of the transgene in the experimental mouse groups (**B**). Immunostaining for MUC5B protein was performed using an anti-MUC5B polyclonal antibody, which cross-reacts with both human and mouse MUC5B (**C**,**D**). Additionally, MUC5B protein staining was conducted using an anti-human MUC5B monoclonal antibody (**E**,**F**). Scale bars indicate 100 µm. Data are expressed as the mean ± SD. Statistical analysis was performed using ANOVA with the Neuman-Keuls test. * *p* < 0.05; *** *p* < 0.001; **** *p* < 0.0001. WT, wild-type; TG, transgenic; BLM, bleomycin; SAL, saline.

Lung fibrosis was induced in wild-type (WT/BLM) and human MUC5B rs35705950 transgenic (h-rs35705950-Tg/BLM) mice through continuous subcutaneous administration of bleomycin (BLM). Control groups, consisting of wild-type (WT/SAL) and human MUC5B rs35705950 transgenic (h-rs35705950-Tg/SAL) mice, received sterile physiological saline. As anticipated, the body weight of the mice that received BLM decreased significantly compared to their respective saline-treated control groups (Figure 3A). Human MUC5B mRNA was exclusively detected in the mice carrying the human *MUC5B* transgene (Figure 3B). MUC5B expression was identified using a polyclonal anti-human MUC5B antibody that cross-reacts with mouse MUC5b. Notably, significant expression of the mucin protein was observed in the epithelial cells of large bronchial segments, but not in the distal bronchiolar or alveolar epithelial cells in the human MUC5B rs35705950 TG mice compared to their WT counterparts (Figure 3C,D). Additionally, immunostaining of human MUC5B was performed using a monoclonal anti-human MUC5B antibody. MUC5B staining was significantly elevated in the h-rs35705950-Tg/BLM group compared to the control h-rs35705950-Tg/SAL group. As expected, human MUC5B protein expression was undetected in the WT/BLM or WT/SAL groups using the anti-human MUC5B monoclonal antibody.

### 3.3. Reduced Infiltration of Inflammatory Cells in Human MUC5B rs35705950 Transgenic Mice with Lung Fibrosis

**Figure 4:**
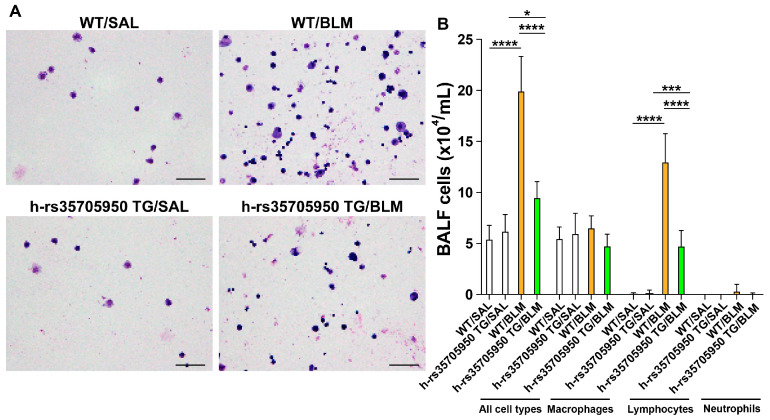
Reduced infiltration of inflammatory cells in human MUC5B rs35705950 transgenic mice with lung fibrosis. (**A**,**B**) Lung fibrosis was induced in wild-type (WT/BLM) and human MUC5B rs35705950 transgenic (h-rs35705950-Tg/BLM) mice through continuous subcutaneous administration of BLM. Control groups, consisting of WT (WT/SAL) and human MUC5B rs35705950 transgenic (h-rs35705950-Tg/SAL) mice, similarly received sterile physiological saline. On the 22nd day following BLM administration, bronchoalveolar lavage fluid was collected under profound anesthesia. The total cell count and differential cell count were then assessed. Scale bars indicate 200 μm. Data are expressed as the mean ± SD. Statistical analysis was performed by ANOVA with Neuman-Keuls test. * *p* < 0.05, *** *p* < 0.001, **** *p* < 0.0001. WT, wild-type; TG, transgenic; BLM, bleomycin; SAL, saline.

On the 22nd day following BLM administration, bronchoalveolar lavage fluid (BALF) was collected under profound anesthesia. The total cell count and differential cell count were then assessed. The total number of all inflammatory cells and lymphocytes was significantly elevated in both WT and MUC5B rs35705950 transgenic mice with lung fibrosis compared to their respective saline control groups. However, these cell counts were markedly lower in the human MUC5B rs35705950 TG mice with lung fibrosis (h-rs35705950-Tg/BLM) than in their WT counterparts (Figure 4A,B). No significant difference was observed in the total counts of inflammatory cells and lymphocytes between WT and TG mice without lung fibrosis. These findings suggest a potential protective role of the human MUC5B rs35705950 transgene against inflammatory cell infiltration in lung fibrosis.

### 3.4. Reduced Expression of Inflammatory Cytokines in Human MUC5B rs35705950 Transgenic Mice with Lung Fibrosis

**Figure 5:**
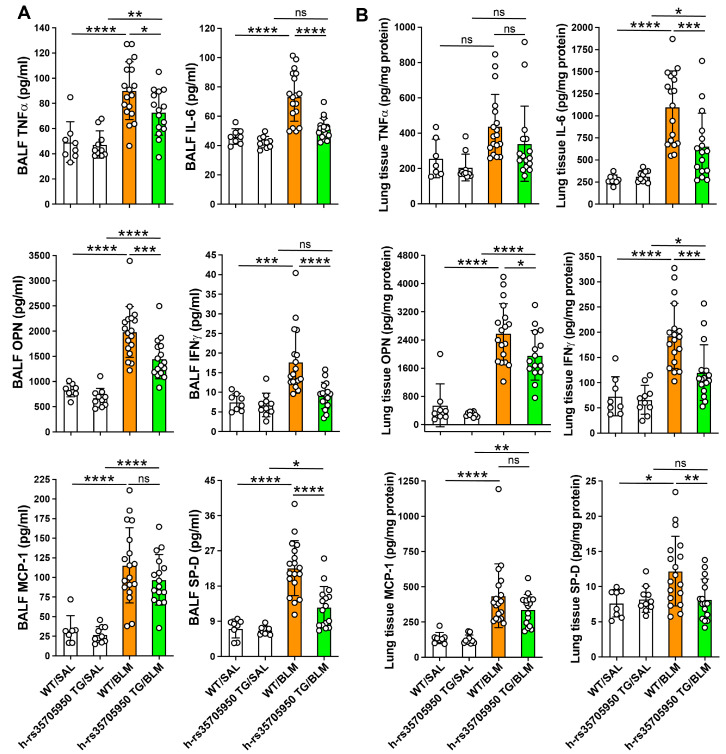
Reduced expression of inflammatory cytokines in human MUC5B rs35705950 transgenic mice with lung fibrosis. Inflammatory cytokines were measured in bronchoalveolar lavage fluid (**A**) and lung tissue homogenate (**B**) by immunoassays using commercially available kits and following the protocols of the manufacturers. Data are expressed as the mean ± SD. Statistical analysis was performed by ANOVA with Neuman-Keuls test. * *p* < 0.05, ** *p* < 0.01, *** *p* < 0.001, **** *p* < 0.0001. WT, wild-type; TG, transgenic; BLM, bleomycin; SAL, saline; ns, not significant; OPN, osteopontin.

Inflammatory cytokine concentrations were quantified using commercially available immunoassay kits in plasma and lung tissue homogenates. Within the BALF, levels of tumor necrosis factor-α (TNFα), osteopontin (OPN), monocyte chemoattractant protein-1 (MCP-1), and surfactant protein-D (SP-D) were significantly elevated in both wild-type (WT) and TG mice with lung fibrosis, compared to their respective saline-treated controls. Additionally, the BALF concentrations of TNFα, interleukin-6 (IL-6), osteopontin, interferon-γ (IFNγ), and SP-D were substantially lower in TG mice with lung fibrosis than in their WT counterparts. No significant differences were noted in the BALF levels of MCP-1 between WT and TG mice with lung fibrosis (Figure 5A).

In the lung tissues, IL-6, osteopontin, IFNγ, and MCP-1 levels were markedly increased in WT and TG mice with lung fibrosis compared to their respective saline-treated controls. The lung tissue levels of IL-6, osteopontin, IFNγ, and SP-D were significantly lower in the TG group with lung fibrosis than in their WT counterparts. There were no statistical differences in the lung tissue levels of MCP-1 between WT and TG mice with lung fibrosis (Figure 5B). These data collectively indicate that the transgenic expression of the human MUC5B rs35705950 variant modulates the inflammatory milieu in the context of lung fibrosis.

### 3.5. Decreased Expression of Growth Factors in Human MUC5B rs35705950 Transgenic Mice with Lung Fibrosis

**Figure 6:**
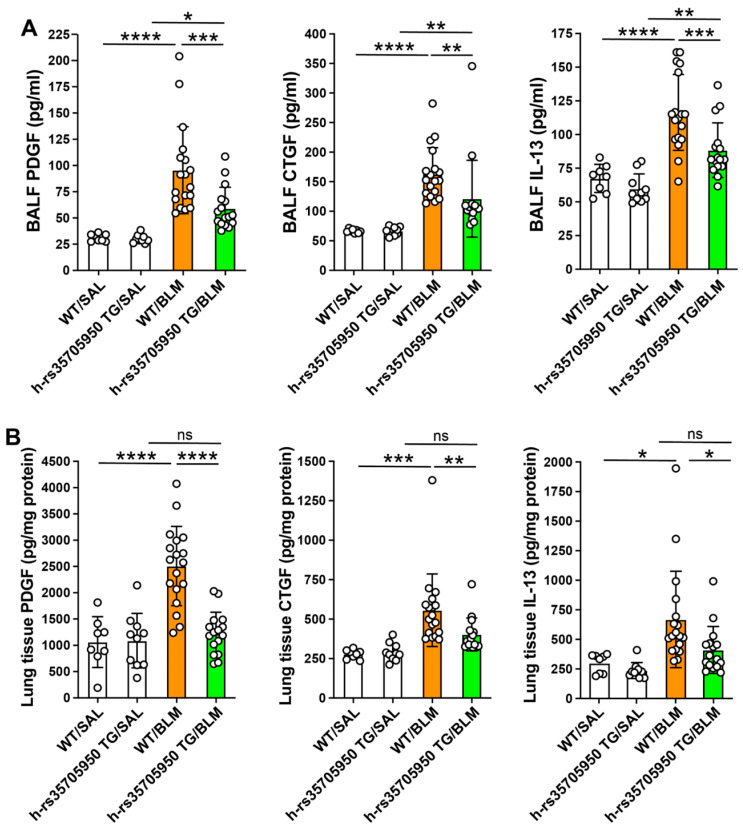
Decreased expression of growth factors in human MUC5B rs35705950 transgenic mice with lung fibrosis. Growth factors were measured in bronchoalveolar lavage fluid (**A**) and lung tissue homogenate (**B**) by immunoassays using commercially available kits and following the protocols of the manufacturers. Data are expressed as the mean ± SD. Statistical analysis was performed by ANOVA with Neuman-Keuls test. * *p* < 0.05, ** *p* < 0.01, *** *p* < 0.001, **** *p* < 0.0001. WT, wild-type; TG, transgenic; BLM, bleomycin; SAL, saline; ns, not significant.

The levels of growth factors were measured using commercially available immunoassay kits. In BALF, the concentrations of platelet-derived growth factor (PDGF), connective tissue growth factor (CTGF), and the profibrotic T helper 2 cytokine IL-13 were significantly elevated in both WT and TG mice with lung fibrosis compared to their respective saline-treated controls. Notably, MUC5B rs35705950 TG mice with lung fibrosis exhibited markedly reduced PDGF, CTGF, and IL-13 levels compared to their WT counterparts (Figure 6A). Additionally, the PDGF, CTGF, and IL-13 concentrations in lung tissue homogenates were significantly higher in WT mice with lung fibrosis than in saline-treated control WT mice and TG mice with lung fibrosis. No significant differences were observed in the concentrations of growth factors between the TG mice with and without lung fibrosis (Figure 6B). These findings suggest differential regulation of growth factors in TG versus WT mice, highlighting the potential protective effects of the MUC5B rs35705950 variant in modulating fibrotic processes.

### 3.6. Reduced Expression of Extracellular Matrix Markers in Human MUC5B rs35705950 Transgenic Mice with Lung Fibrosis

**Figure 7:**
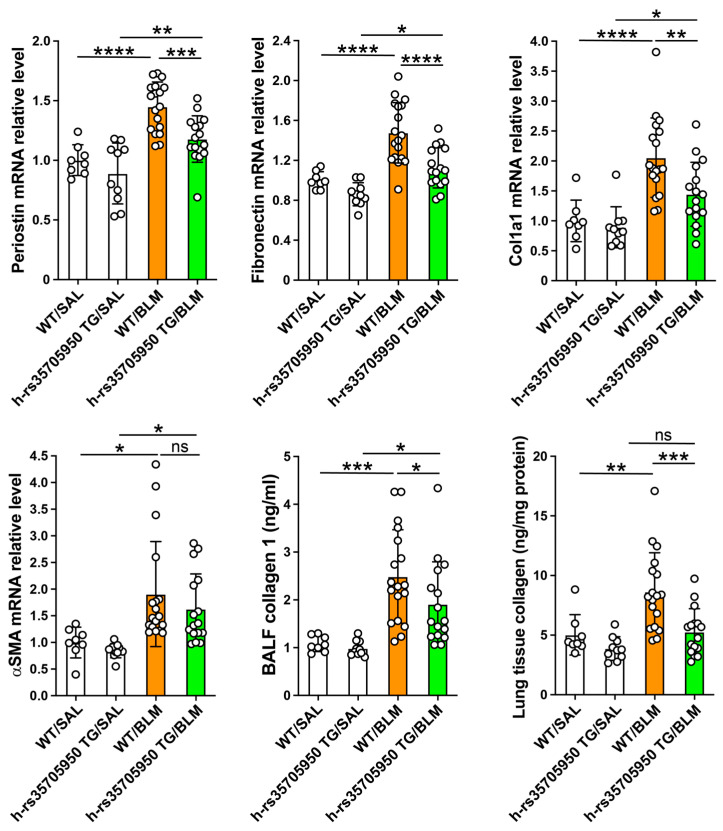
Reduced expression of extracellular matrix markers in human MUC5B rs35705950 transgenic mice with lung fibrosis. The relative mRNA expression of extracellular matrix markers was assessed by polymerase-chain reaction and the levels of collagen I was assessed by enzyme immunoassays using commercially available kits following the protocol of the manufacturers. Data are expressed as the mean ± SD. Statistical analysis was performed by ANOVA with Neuman-Keuls test. * *p* < 0.05, ** *p* < 0.01, *** *p* < 0.001, **** *p* < 0.0001. WT, wild-type; TG, transgenic; BLM, bleomycin; SAL, saline; ns, not significant.

The relative mRNA expressions of periostin, fibronectin, collagen type I alpha 1 (Col1a1), and alpha-smooth muscle actin (α-SMA) were significantly elevated in both WT and TG mice with lung fibrosis compared to their respective saline-treated controls. Notably, the expressions of periostin, fibronectin, and Col1a1 were significantly reduced in rs35705950 TG mice with lung fibrosis relative to their WT counterparts. However, the mRNA expression of α-SMA showed no significant differences between the WT and TG mice with lung fibrosis (Figure 7). Additionally, the levels of collagen I in BALF and lung tissue homogenates were significantly higher in WT mice with lung fibrosis compared to saline-treated control WT mice and rs35705950 TG mice with lung fibrosis (Figure 7). These results demonstrate a differential modulation of fibrosis-related genes and collagen production between WT and rs35705950 TG mice, suggesting a potential protective effect of the rs35705950 mutation against lung fibrosis development.

### 3.7. Decreased Lung Fibrosis in Human MUC5B rs35705950 Transgenic Mice

**Figure 8:**
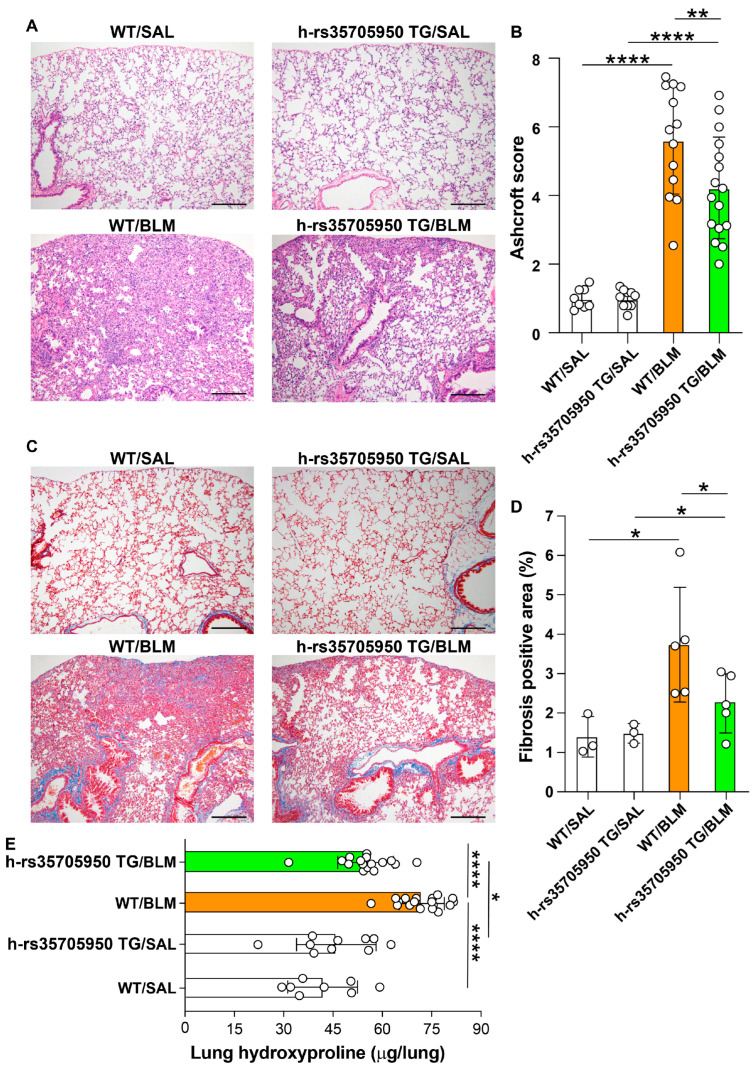
Reduced lung fibrosis in human MUC5B rs35705950 transgenic mice. Ashcroft scoring was performed in lung tissue stained with hematoxylin & eosin by blinded experts for the treatment groups. The number of mice in: WT/SAL, n = 8, WT/BLM, n = 18, h-rs35705950-TG/SAL, n = 10, h-rs35705950-TG/BLM, n = 16 (**A**,**B**). Collagen deposition was evaluated after trichrome staining, and the total lung collagen volume fraction was calculated. The number of mice: WT/SAL, n = 3, WT/BLM, n = 5, h-rs35705950-TG/SAL, n = 3, h-rs35705950-TG/BLM, n = 5 (**C**,**D**). The lung tissue hydroxyproline content was measured by a colorimetric assay. The number of mice: WT/SAL, n = 8, WT/BLM, n = 18, h-rs35705950-TG/SAL, n = 10, h-rs35705950-TG/BLM, n = 16 (**E**). Scale bars indicate 200 µm. Data are the mean ± S.D. Statistical analysis by ANOVA with Newman-Keuls test. * *p* < 0.05, ** *p* < 0.001; **** *p* < 0.001. WT, wild-type; SAL, saline; BLM, bleomycin.

The severity of lung fibrosis in mice was evaluated using trichrome and hematoxylin and eosin (H&E) staining, Ashcroft scoring, and measurement of lung hydroxyproline content. Both the area of fibrosis, as determined by trichrome staining, and the fibrosis grade, as assessed by the Ashcroft score, along with lung hydroxyproline levels, were significantly increased in WT and MUC5B rs35705950 TG mice treated with BLM, compared to their respective saline-treated control groups. Conversely, the area of fibrosis, the Ashcroft score, and the lung hydroxyproline content were significantly lower in TG mice with lung fibrosis than in their WT counterparts (Figure 8A–E). These findings suggest that the MUC5B rs35705950 transgene imparts a protective effect against BLM-induced lung fibrosis in mice, evidenced by reduced fibrosis severity and lower collagen deposition in transgenic mice relative to WT controls.

## 4. Discussion

This study demonstrates that the overexpression of mucin in the proximal airways, associated with the human *MUC5B* [rs35705950] T allele mutation, confers protection against inflammation and fibrosis in the mouse model of BLM-induced pulmonary fibrosis.

Mucins, the high molecular weight glycoproteins secreted by the epithelial linings of the respiratory tract, play a critical role in pulmonary defense under normal physiological conditions [21,29,30]. These glycoproteins are vital for maintaining the integrity and functionality of the pulmonary barrier, shielding against microbial invasion, and aiding the clearance of inhaled particulates via the mucociliary transport system [21,31,32,33]. However, dysregulated production of mucins, whether excessive or ectopic, is associated with the pathogenesis and exacerbation of various lung diseases [34,35]. In chronic obstructive pulmonary disease (COPD), mucins bolster mucociliary clearance, facilitating the expulsion of pathogens and reducing the risk of infections that can exacerbate the condition [36,37]. However, mucin overproduction, especially evident in the chronic bronchitis phenotype of COPD, can lead to substantial airway obstruction, increasing both morbidity and mortality [36,37]. Similarly, in bronchial asthma, while elevated mucin levels can trap allergens and pollutants, preventing their more profound penetration into the lungs, an excess can obstruct airways, leading to wheezing and breathing difficulties [38,39,40]. Mucins also play a complex role in interstitial lung diseases and lung fibrosis, where they can be both protective and detrimental [20]. Optimal mucin levels act as physical barriers and promote immune tolerance, potentially protecting against lung fibrosis [35,39,41]. Conversely, aberrant expression of specific mucins like MUC1 and MUC5B may contribute to fibrogenesis by impairing mucociliary clearance and facilitating the retention of pollutants, cigarette smoke toxins, microorganisms, and inflammatory debris [20,42]. While mucin overexpression in the distal airways, including terminal and respiratory bronchioles and alveoli, is known to promote lung fibrosis, the potential protective effects of mucin overexpression in the proximal airways remain unexplored [11]. The current study investigates whether mucin overproduction is associated with the human *MUC5B* [rs35705950] variant in the proximal airways, conferring protection against BLM-induced lung fibrosis in mice.

The *MUC5B* promoter polymorphism rs35705950 is recognized as the most potent genetic predisposing factor for both familial and sporadic forms of IPF [6,7,8,9]. In healthy individuals, MUC5B expression is typically restricted to the bronchi and proximal bronchioles. However, in IPF patients, enhanced expression of MUC5B is observed in the epithelium of honeycombing cysts and within intra-cystic mucus plugs [8,11,12]. Further research has confirmed the co-expression of MUC5B and surfactant protein C, an alveolar epithelial type II cell marker, in the epithelial cells lining the honeycombing cysts of IPF lung tissue [11]. This pathological feature has been replicated in transgenic mice models, where overexpression of MUC5b in the alveoli and/or distal bronchioles mirrors these human IPF observations [11]. Pre-clinical studies utilizing these transgenic mice have shown that they develop significantly more severe lung fibrosis than their WT counterparts, which typically express MUC5b in the larger bronchial airways [11]. This evidence highlights the pivotal role of MUC5B overexpression in the alveolar and distal bronchiolar compartments in IPF pathogenesis.

Interestingly, despite the adverse effects of MUC5B overexpression, IPF patients harboring the rs35705950 polymorphism exhibit more prolonged survival, slower disease progression, reduced lung bacterial burden, less severe pathological changes, and a slower decline in forced vital capacity compared to non-carriers [2,10,14,16,17,18,19]. To address this paradox, our current study assessed whether localized overexpression of the MUC5B rs35705950-related mucin in large bronchial segments and proximal bronchioles, without extending to distal bronchioles or alveoli, might confer a protective effect. Our findings with a human *MUC5B* [rs35705950] variant TG mouse model provide compelling evidence that mucin overexpression in the large bronchi and proximal bronchioles plays a protective role in the respiratory system. Specifically, we observed that MUC5B overexpression can significantly protect against inflammation, reduce the expression of inflammatory cytokines and growth factors, and mitigate the development of lung fibrosis in a bleomycin-induced model of lung fibrosis in mice. These results align with the well-established role of MUC5B in mucociliary clearance, a critical defense mechanism that ensures the removal of pathogens and other harmful particulates from the airways. For instance, Thornton et al. reported that the mucin barrier is essential for trapping and expelling pathogens, thereby preventing infection and maintaining respiratory health [21]. Similarly, Knowles and Boucher demonstrated that efficient mucin secretion is a primary innate defense mechanism crucial to preventing chronic airway diseases, while Roy et al. showed that the absence of MUC5B severely compromises bacterial clearance in the lungs [43,44]. The protective role of MUC5B overexpression observed in our study not only supports these previous findings but also provides new insights into the potential therapeutic benefits of modulating mucin levels to prevent or treat lung fibrosis and other related conditions. Furthermore, these findings highlight the importance of the specific localization of mucin overexpression in influencing the pathogenesis of lung fibrosis.

## 5. Conclusions

This investigation underscores the significance of mucin overexpression at distinct pulmonary locations, mediated by the *MUC5B* promoter polymorphism rs35705950, in modulating the progression of pulmonary fibrosis. While the extant literature identifies the enhancement of lung fibrosis through MUC5B overexpression within the alveolar and distal bronchiolar compartments, our findings reveal that targeted overexpression within the proximal airways may impart protective advantages. This finding highlights the importance of the specific localization of MUC5B activity in influencing disease progression. The research thus offers valuable insights into potential therapeutic strategies that could selectively modulate MUC5B expression to mitigate the progression of fibrotic processes in the lungs.

## Figures and Tables

**Table 1:** Primers for RT-PCR.

Gene & Direction	Sequence (5′ to 3′)	Length (nt)	Tm (°C)	Accession Number	Position	Product
hMUC5B						
Forward	GCCCACATCTCCACCTATGAT	21	61.1	NM_002458.3	1349-1369	141 bp
Reverse	GCAGTTCTCGTTGTCCGTCA	20	62.4		1489-1470	
mMuc5B						
Forward	TCCCTAGCATGAGCGCCTTA	20	62.6	NM_028801.2	197-216	178 bp
Reverse	CCACGACGCAGTTGGATGTT	20	63		374-355	
mPeriostin						
Forward	CACGGCATGGTTATTCCTTCA	21	60.4	NM_001198766.1	547-567	151 bp
Reverse	TCAGGACACGGTCAATGACAT	21	61.1		697-677	
mFibronectin						
Forward	TTCAAGTGTGATCCCCATGAAG	22	60	NM_010233.2	7126-7147	154 bp
Reverse	CAGGTCTACGGCAGTTGTCA	20	61.5		7279-7260	
mCollagen I						
Forward	TAAGGGTCCCCAATGGTGAGA	21	63.8	NM007742.4	107-127	203 bp
Reverse	GGGTCCCTCGACTCCTACAT	20	60.3		309-290	
mα-SMA						
Forward	CAGGATGCAGAAGGAGATCAC	21	60.7	NM007392.2	1009-1029	364 bp
Reverse	TGTTGCTAGGCCAGGGCTAC	20	62.6		1372-1353	
mGapdh						
Forward	TGGCCTTCCGTGTTCCTAC	19	61.3	NM_008084.4	686-704	178 bp
Reverse	GAGTTGCTGTTGAAGTCGCA	20	60.9		863-844	
Gapdh: glyceraldehyde 3-phosphate dehydrogenase; αSMA, αsmooth muscle actin; h, human; m, mouse.						

## Data Availability

The authors declare that all data supporting this study’s findings are available within this manuscript.
